# Biochemical Characterization and Antimicrobial Properties of Extracts From Four Food Plants Traditionally Used to Improve Drinking Water Quality in Rural Areas of Burkina Faso

**DOI:** 10.1155/bri/9994531

**Published:** 2025-02-18

**Authors:** Frédéric Anderson Konkobo, Poussian Raymond Barry, Sandrine Zongo, Elisabeth Rakisewendé Ouédraogo, Noëlle Edwige Roamba, Roger Dakuyo, David Bazié, Balamoussa Santara, Mamounata Diao, Paul Windinpsidi Savadogo, Mamoudou Hama Dicko

**Affiliations:** ^1^Laboratory of Biochemistry, Biotechnology, Food Technology and Nutrition (LABIOTAN), Department of Biochemistry, and Microbiology, University Joseph Ki-Zerbo, Ouagadougou 03 BP 7021, Burkina Faso; ^2^Department of Biochemistry and Microbiology, Training and Research Unit in Life and Earth Sciences, Nazi Boni University, Bobo-Dioulasso, Burkina Faso; ^3^Department of Natural Resource Management and Production Systems, Soil-Water-Plant Laboratory, Institute of Environment and Agricultural Research/National Center for Scientific and Technological Research (INERA/CNRST), Ouagadougou 01 BP 476, Burkina Faso

**Keywords:** antimicrobial activity, biochemical characterization, drinking water, food plants, quality

## Abstract

The use of local plant extracts in drinking water purification represents a sustainable alternative in the fight against diseases linked to unsafe water consumption in rural areas. The aim of this study was to evaluate the biochemical composition and antimicrobial activity of four local plant extracts used in rural areas of Burkina Faso to purify drinking water: *Moringa oleifera* seeds, *Boscia senegalensis* seeds, *Opuntia ficus-indica* cladodes, and *Aloe vera* leaves. These four extracts were then subjected to biochemical screening to identify phytocompounds, followed by quantification and evaluation of their antibacterial properties on ten pathogenic bacterial strains. The screening results revealed the presence of a variety of molecules (phenolic compounds, alkaloids, saponosides, etc.) in the different extracts studied. From a quantitative point of view, *M. oleifera* and *B. senegalensis* seeds showed a high total protein content (34.5 and 24.6 g/100 g DM). *A. vera* and *O. ficus-indica* extracts showed high levels of total carbohydrates (20.4 and 35.52 g/100 g DM) compared with total lipids and proteins. The same applies to phenolic compounds, which were also high in *A. vera* and *O. ficus-indica* extracts (17.42 and 26.5 mg GAE/100 mg DM) compared to *M. oleifera* and *B. senegalensis* seeds. In terms of antibacterial properties, the four extracts studied showed inhibition diameters ranging from 7.33 to 16.33 mm. These results reflect the ability of the different extracts to eliminate pathogenic microorganisms present in water. Overall, this study stands out for its innovative character, offering an in-depth understanding of the biochemical composition and antimicrobial properties of four distinct extracts of local plants commonly used in traditional drinking water purification practices. It enriches existing knowledge by providing new data on the biochemical composition and bioactivity of these extracts. In particular, the study highlights the synergistic effects of the bioactive compounds present, underlining their essential role in improving the sanitary quality of water consumed in rural areas, where sustainable and accessible solutions are crucial.

## 1. Introduction

Access to drinking water is a fundamental requirement for human health and well-being. However, in many regions of the world, particularly in rural areas and poor communities, clean drinking water remains woefully inaccessible [1, 2]. This situation exposes local populations to a considerable risk of contracting fatal waterborne diseases such as dysentery and cholera [3]. Faced with this public health problem, the use of local plant extracts in the treatment of drinking water appears to be a traditional solution that merits in-depth study and scientific validation. Indeed, for centuries, indigenous plants have been traditionally used to improve water quality in many communities of African and Latin American [4]. Moreover, in recent years, a few scientific studies have explored the biopurifying capacity of various local food plant extracts based on seeds, leaves, stems, husks, and fruits [5]. Among the many plant extracts studied, *M. oleifera* and *B. senegalensis* seeds, *A. vera* leaves and *O. ficus-indica* cladodes showed excellent performance in the treatment of drinking water [6]. In fact, these extracts, prepared as aqueous solutions, act as excellent biocoagulants and enable the potabilization of any type of raw water, regardless of its degree of turbidity [7].

However, the biocoagulant properties of these plant extracts in water treatment suggest that they contain bioactive compounds, responsible for their purifying and antimicrobial activities [8, 9]. Unfortunately, few studies have been carried out on this subject, and there is little scientific information available on the biochemical composition and antimicrobial activity of these 04 plant extracts, which are nevertheless highly effective in water treatment. It therefore seems essential to gain an in-depth understanding of the composition and diversity of the various compounds in these plant extracts, in order to assess their efficacy and integrate them rationally into community water treatment practices in rural areas.

This study will therefore establish a link between the traditional use of these plant extracts and their actual effectiveness in water treatment, opening up prospects for innovative and sustainable approaches aimed at improving access to safe drinking water for local populations.

This is the context of our study, the aim of which is to evaluate the chemical composition and antimicrobial activity of *M. oleifera*, *B. senegalensis*, *A. vera*, and *O. ficus-indica* extracts traditionally used in the treatment of drinking water. More specifically, the first step was to identify and analyze the bioactive compounds present in the 4 plant extracts studied; the second step was to evaluate their antibacterial activity against potentially harmful strains of food-borne microorganisms.

## 2. Materials and Methods

### 2.1. Plant Material

**Figure 1:**
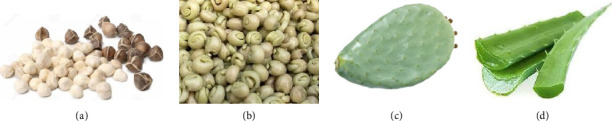
*M. oleifera* seeds (a); *B. senegalensis* seeds (b); *O. ficus-indica* (c); *A. vera* (d).

The food plant extracts studied were mainly *M. oleifera* and *B. senegalensis* seeds, as well as *A. vera* and *O. ficus-indica* cladodes (Figure 1). *M. oleifera* seeds were collected in Garango with the technical support of the National Center of Forest Seeds of Burkina Faso (NCFS) in March 2022. *B. senegalensis* seeds, *A. vera* leaves, and *O. ficus*-indica cladodes were collected in Ouagadougou's “Bangr weeogo” urban park in May 2022.

### 2.2. Microbiological Material

The microorganisms used to assess the antibacterial activity of the various plant extracts were supplied by the Department of Food Technology of the National Center for Scientific Research and Technology (DFT/NCSRT) and the Laboratory of Biochemistry, Biotechnology, Food Technology and Nutrition (LABIOTAN) in Burkina Faso. The Gram-positive bacterial reference strains were *Bacillus subtilis* ATCC 25923; *Bacillus cereus* 13569; *Micrococcus luteus* SKN 624; *Listeria monocytogenes* NCTC 9863; and *Staphylococcus aureus* ATCC 252. Gram-negative bacterial strains were *Escherichia coli* 25922; *Yersinia enterocolitica* 8A30 SKN 601; *Pseudomonas aeruginosa* ATCC 9027; *Shigella dysenteriae* SKN 557; and *Salmonella typhimurium*.

### 2.3. Biochemical Composition

#### 2.3.1. Biochemical Screening

Biochemical screening was carried out to identify compounds such as polyphenols, flavonoids, tannins, saponosides, alkaloids, and terpenes in the 4 extracts studied. To this end, a 500 g/L ethanolic stock solution of each extract was prepared after maceration for 48 h and then used as the crude extract for the various highlights [10]. Thus,

• For flavonoid screening, 3 mL of crude extract was evaporated. The residue was then dissolved in 2 mL methanol 50%. A fragment of magnesium turnings was then added, followed by 5 drops of concentrated hydrochloric acid. The appearance of a red or orange coloration in the solution thus indicates the presence of flavonic aglycones in the extracts [11].
• For tannin screening, 1 mL crude extract was diluted with 2 mL distilled water, and then 2 drops of 1% FeCl_3_ solution were added to the mixture. The appearance of a blue-black coloration indicates the presence of gallic tannins, while a blackish-green coloration indicates the presence of catechic tannins [11].
• For saponoside screening, 2 mL of crude extract, diluted by ½, was shaken in a test tube for 15 min. The appearance of a 1-cm column of foam persisting for at least 15 min indicates the presence of saponosides [12].
• For alkaloid screening, 5 mL of crude extract was added to 50 mL of sulfuric acid diluted 1/20, then left to macerate for 2 hours. Five drops of Dragendorff's reagent were then added to 1 mL of the solution. A positive result was expressed by the appearance of a precipitate [12].

#### 2.3.2. Quantitative Determination of Phytocompounds

##### 2.3.2.1. Preparation of Methanolic Extracts

Methanolic extracts were prepared by dispersing 500 mg of the previously ground and dried powder of each sample in 15 mL of 80% (v/v) methanol under continuous stirring at 200 rpm for 4 h at 25°C. After centrifugation at 4000 rpm, the supernatant is the crude extract used for analysis.

##### 2.3.2.2. Determination of Phenolic Compounds Content

Total phenolic content (TPC) was determined using the Folin–Ciocalteu method [13]. It is based on the high oxidizability of phenolic compounds. The colorimetric properties of the Folin–Ciocalteu reagent (FCR) are modified when it is complexed with certain molecules. It reacts with the OH function of phenols. Absorbance was read at 760 nm with a spectrophotometer. The TPC was expressed as mg gallic acid equivalent (mg GAE) per 100 mg of dry matter (DM) [14].

##### 2.3.2.3. Determination of Flavonoids

The flavonoids content was determined using the colorimetric method with aluminum trichloride (AlCl_3_) [13]. This method is based on the aluminum chloride (AlCl_3_) test. A 0.5 mL of methanol of each extract solution (0.1 mg/mL) was mixed with 1.5 mL of AlCl_3_ (2%) and incubated for 30 min at room temperature after this incubation period. Absorbance was read spectrophotometrically against a blank at 415 nm. Results were expressed as milligrams of quercetin equivalent (mg QE) per 100 mg of DM [14].

##### 2.3.2.4. Determination of Tannin Content

The hydrolyzable tannin content was determined using the ferric trichloride (FeCl_3_) method [10]. This consisted of adding 1 mL of each extract (5 mg/mL) to 3.5 mL of a solution prepared from 0.01 M ferric trichloride (FeCl3) in 0.001 M hydrochloric acid (HCl). After 15 s, the absorbance of the mixture was read at 660 nm. The results were expressed as mg GAE per gram of dry extract (mg GAE/100 mg) [14].

##### 2.3.2.5. Protein Determination

The protein content was determined by the Bradford method [15] with minor modifications. This involved homogenizing 500 mg of powder from each sample in 10 mL of an extraction solution (0.1 M NaCl). The mixture was centrifuged at 4500 rpm for 15 min, and the supernatant collected was used for protein assay. A volume of 50 μL of extract was added to 250 μL of Bradford reagent. Optical density was determined using a spectrometer at 595 nm against a blank consisting of 50 μL extract and 250 μL buffer solution.

##### 2.3.2.6. Determination of Total Carbohydrates

The total soluble carbohydrates were determined using the phenol–sulfuric acid method [10]. This consisted of mixing an aliquot of 100 mg of each sample with 3 mL of 80% ethanol and leaving it at room temperature for 48 h. The ethanol was then evaporated using a water bath at 100°C. After evaporation, 20 mL of distilled water was added to the dry residue. Next, 4 mL of anthrone reagent was added to a test tube containing 2 mL of the obtained extract, and the mixture was heated in a water bath at 62°C for 8 min. After cooling in an ice bath, the tube was allowed to rest in the dark for 30 min, and the absorbance was measured at 490 nm by spectrophotometry.

##### 2.3.2.7. Determination of Total Lipids

Total lipids' quantification was performed using the Soxhlet extraction method [15]. This consisted of placing the sample in a cellulose cartridge, which was then inserted into the Soxhlet apparatus. The flask was weighed and filled with hexane, and the extractor was assembled with a condenser. At least ten extraction cycles were performed. A 10-g test sample of each was used to obtain lipid content.

##### 2.3.2.8. Determination of Ash, Moisture, and DM Content

Ash content was determined using the AFNOR method. For each sample, 5 g of ground powder was placed in a muffle furnace set at 550 ± 15°C for 5 h until a gray, clear, or whitish color was obtained. Ash content was calculated on the basis of mass difference.

Moisture content and DM were determined using the thermogravimetric method [10]. This involved introducing 5 g of each sample into a crucible, which was then placed in an oven maintained at 105°C until a constant weight was obtained. The crucible was then cooled in a desiccator for 30 min and weighed again. The difference in weight before and after oven-drying was used to calculate moisture content.

The DM content was deduced from the moisture content using the following formula: % DM = 100% − %H, where DM represents dry matter content; and H represents moisture content.

### 2.4. Assessing the Antimicrobial Properties of Biocoagulants

#### 2.4.1. Preparation of Strains, Inoculum, and Cell Culture Media

The antibacterial activity of the various extracts was determined using the disk diffusion method [13] with slight modifications. To prepare the inoculum, the various bacterial strains were streaked onto Petri dishes containing nutrient agar. These were then incubated at 37°C for 24 h to obtain isolated colonies. After incubation, isolated colonies of the bacterial species concerned were suspended in NaCl solution (0.9%), and then adjusted to an optical density of 0.5 Mac Farland standards (10^8^ CFU/mL). To obtain inoculum, the suspensions were diluted 100-fold in MH broth to give 10^6^ colony-forming units (CFU)/mL.

#### 2.4.2. Preparation of Culture Media

The Mueller–Hinton agar medium (MH) was obtained by dissolving 38 g of MH medium in 1 L of distilled water (pH = 7.5 ± 0.2), and the MH broth by dissolving 21 g in 1 L of distilled water. Each medium was sterilized in an autoclave at a temperature of 120°C for approximately 15 min. After sterilization, 10 mL of the Mueller–Hinton agar was poured into sterile 90-mm-diameter Petri dishes. The agar thickness was 2 mm, evenly spread in the dishes. The dishes were allowed to dry at room temperature for 30 min before use.

#### 2.4.3. Disk Preparation

Ethanolic extracts were dissolved in DMSO to give final concentrations of 100 and 200 mg/L. Each stock solution was then sterilized by filtration through a sterilizing filter (0.22 μm). Sterile disks (6 mm) were impregnated with 10 μL of each solution. A negative control was prepared using disks impregnated with DMSO and a positive control from disks impregnated with the antibiotic gentamicin [13].

#### 2.4.4. Determination of Zone of Inhibition (ZOI) Diameters

Petri dishes previously prepared from MH agar were inoculated with 15 μL of each bacterial suspension (10^6^ CFU/mL). After drying in a sterile hood, 6-mm-diameter disks soaked with 10 μL of ethanolic solutions of the different extracts were then placed on the agar. Disks soaked in gentamicin and DMSO were used as positive and negative controls, respectively. Petri dishes were then incubated at 37°C for 24 h. Antibacterial activity was determined according to the diameter of the ZOI produced around the disks, using a graduated ruler. Each test was carried out in triplicate, and the strain sensitivity was assessed using the reading grid [16] (Table 1).

### 2.5. Statistical Analysis

Data were analyzed statistically using XLSTAT software (Addinsoft, 2021). Analyses of variance (ANOVA) were performed, with Tukey's test at the 5% threshold for comparison of means, as well as principal component analysis (PCA). All experimental measurements were carried out in at least triplicate. Results were expressed as mean ± standard error (SD) of the mean.

## 3. Results and Discussion

### 3.1. Results

#### 3.1.1. Qualitative Analysis of Phytocompounds: Biochemical Screening

Biochemical screening of ethanolic extracts from *M. oleifera*, *B. senegalensis*, *A. vera*, and *O. ficus-indica* revealed the presence of 06 families of secondary metabolites. These are mainly polyphenols, flavonoids, tannins, alkaloids, saponosides, and triterpenes. From a qualitative point of view, the presence of each of these chemical groups was observed in the different extracts studied, with the exception of saponosides in *M. oleifera* extract and alkaloids in *A. vera* extract. The results of these tests are presented in Table 2.

#### 3.1.2. Quantitative Analysis

##### 3.1.2.1. Moisture, DM, and Ash Content

**Figure 2:**
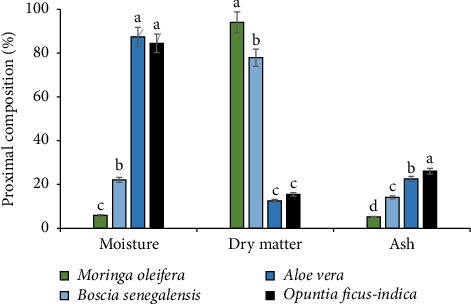
Moisture, dry matter, and ash contents of *M. oleifera* seeds, *B. senegalensis* seeds, *A. vera* leaves, and *O. ficus-indica* cladodes.

The moisture content of *M. oleifera* seeds, *B. senegalensis*, *A. vera* leaves, and *O. ficus-indica* cladodes ranged from 5.9 ± 0.26% to 87.4 ± 1.17%. The highest contents were observed in *O. ficus-indica* cladodes and *A. vera* leaves, compared with *M. oleifera* and *B. senegalensis* seeds, which showed lower contents (Figure 2).

In contrast to moisture, DM content was higher in *M. oleifera* seeds (94.1%) and *B. senegalensis* seeds (77.9%), whereas in *O. ficus-indica* cladodes (15.5%) and *A. vera* leaves (12.6%), it was low.

Ash content was 5.22 ± 0.39% for *M. oleifera* seeds, 14.1 ± 0.87% for *B. senegalensis* seeds, 26.1 ± 1.26% for *O. ficus-indica* cladodes, and 22.58 ± 1.35% for *A. vera* leaves.

##### 3.1.2.2. Macronutrient Content

**Figure 3:**
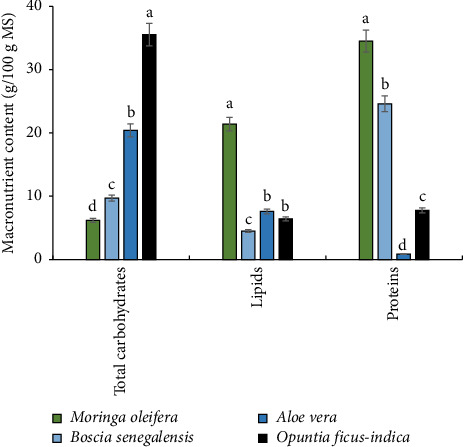
Macronutrient composition of *M. oleifera* seeds, *B. senegalensis* seeds, *A. vera* leaves, and *O. ficus-indica* cladodes.

The macronutrient content, that is, total carbohydrates, lipids, and proteins, of the 04 plant extracts is shown in Figure 3. The results show a variation in total carbohydrates content for each extract, that is, 6.2 ± 0.30 g/100 g DM for *M. oleifera* seeds; 9.7 ± 0.36 g/100 g DM for *B. senegalensis* seeds; 20.4 ± 1.32 g/100 g DM for *A. vera* leaves; and 35.52 ± 1.63 g/100 g DM for *O. ficus-indica* cladodes. The same applies to lipids, with *M. oleifera* and *B. senegalensis* seeds showing contents of 21.4 ± 0.85 g/100 g DM and 4.5 ± 0.51 g/100 g DM, respectively, while *A. vera* leaves and *O. ficus-indica* cladodes showed contents of 7.6 ± 0.45 and 6.42 ± 0.43 g/100 g DM, respectively. Protein content also varied from one extract to another and was 34.5 ± 1.53 g/100 g MS for *M. oleifera* seeds; 24.6 ± 1.72 g/100 g DM for *B. senegalensis* seeds; 0.85 ± 0.09 g/100 g DM for *A. vera* leaves; and 7.78 ± 0.27 g/100 g DM for *O. ficus-indica* cladodes.

##### 3.1.2.3. Phenolic Compounds, Flavonoids, and Tannin Content

**Figure 4:**
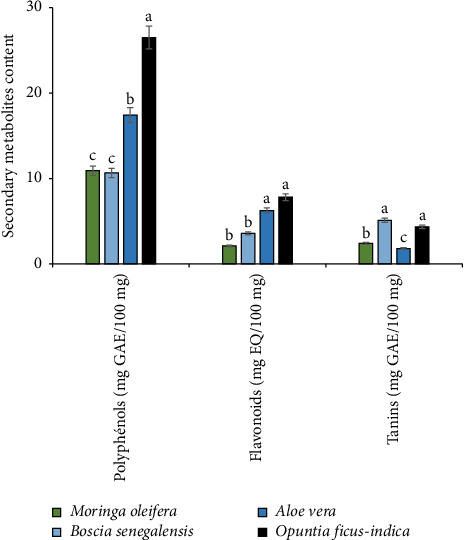
Phenolic compound contents of *M. oleifera* seeds, *B. senegalensis*, *A. vera* leaves, and *O. ficus-indica* cladodes.

Figure 4 shows the content of phenolic compounds (expressed as mg GAE/100 mg DM), flavonoids (mg QE/100 mg DM), and tannins (mg GAE/100 mg DM) for the four plant extracts studied. The results show a significant variation in the content of these different secondary metabolites according to each extract. Total polyphenol content was, respectively, 10.9 ± 1.39; 10.64 ± 1.44; 17.42 ± 1.39; and 26.5 ± 0.81 mg GAE/100 mg DM for *M. oleifera* seeds, *B. senegalensis*, *A. vera* leaves, and *O. ficus-indica* cladodes.

In terms of flavonoids, seeds were found to contain lower levels than leaves and cladodes. *M. oleifera* and *B. senegalensis* seed extracts contained 2.1 ± 0.40 mg QE/100 mg DM and 3.56 ± 1.16 mg QE/100 mg DM, respectively. *A. vera* leaves and *O. ficus-indica* cladodes had mean contents of 6.24 ± 1.67 and 7.8 ± 0.36 mg EQ/100 mg DM, respectively. As for tannins, *M. oleifera* and *B. senegalensis* seeds, *A. vera* leaves, and *O. ficus-indica* cladodes showed relatively low levels of 2.4 ± 0.47; 5.1 ± 0.35; 1.8 ± 0.25; and 4.3 ± 0.43 mg GAE/100 mg DM, respectively.

#### 3.1.3. Antimicrobial Activity of Biocoagulant and Bioflocculant Extracts

##### 3.1.3.1. Inhibition Diameters for Gram-positive Bacterial Strains

**Figure 5:**
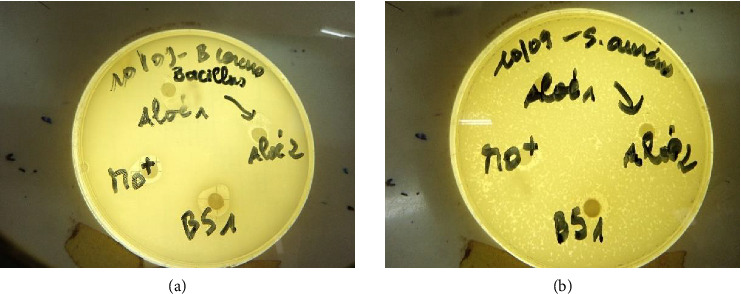
Inhibition diameters of extracts studied on *Bacillus cereus* (a) and *Staphylococcus aureus* (b).

The antibacterial activity of biocoagulant and bioflocculant extracts was tested on 10 bacterial strains, including 5 Gram-positive strains. These 5 strains were *Bacillus cereus*, *Bacillus subtilis*, *Listeria monocytogenes*, *Micrococcus luteus*, and *Staphylococcus aureus*. Each of these strains was sensitive to the action of *M. oleifera*, *B. senegalensis*, *A. vera*, and *O. ficus-indica* extracts at extract concentrations of 100 and 200 mg/mL (Figure 5). Inhibition diameters ranged from 8.00 to 9.67 mm for concentrations of 100 mg/mL and from 12.00 to 16.33 mm for concentrations of 200 mg/mL (Table 3). The largest inhibition diameter (16.33 mm) was observed on the *Listeria monocytogenes* strain for *A. vera* extract (200 mg/mL). The smallest inhibition diameter was 8.00 mm, observed with 100 mg/mL *B. senegalensis* extract on the *Bacillus cereus* strain.

##### 3.1.3.2. Inhibition Diameters for Gram-Negative Bacterial Strains

**Figure 6:**
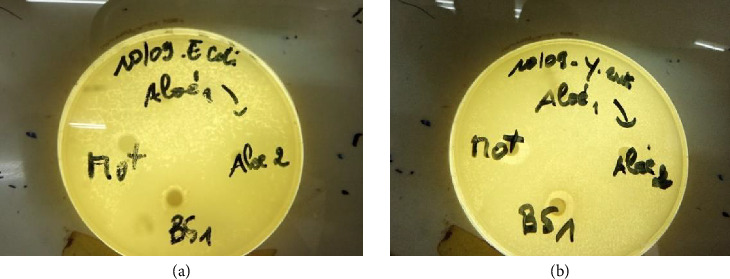
Inhibition diameters of extracts studied on *Escherichia coli* (a) and *Yersinia enterocolitica* (b).

Gram-negative bacterial strains included *Escherichia coli*, *Pseudomonas aeruginosa*, *Salmonella typhimurium*, *Shigella dysenteriae*, and *Yersinia enterocolitica*. Each of these strains also showed sensitivity to the action of the four extracts at concentrations of 100 and 200 mg/mL (Figure 6). Thus, for the 100 mg/mL concentration, the smallest inhibition diameter was 7.33 mm and was observed on the *Salmonella typhimurium* strain after application of *B. senegalensis* extract, while the largest inhibition diameter was 9.33 mm and was observed on the *Shigella dysenteriae* strain after application of *A. vera* extract. For the 200 mg/mL concentration, the smallest inhibition diameter was observed on the *Salmonella typhimurium* strain (11.67 mm) for *A. vera* extract, while the largest inhibition diameter was 14.67 mm and was observed on the *Escherichia coli* strain after application of *M. oleifera* extract (Table 4).

##### 3.1.3.3. PCA

**Figure 7:**
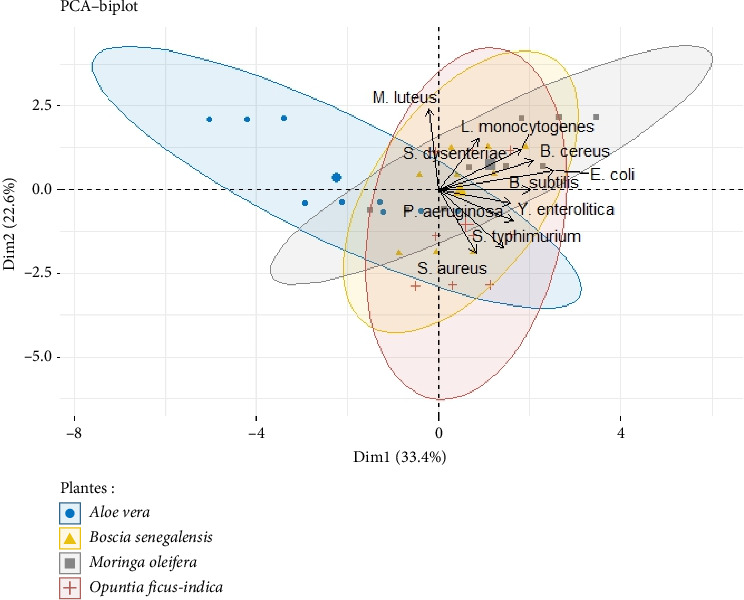
Grouping of inhibition diameters of some bacterial strains according to the bioextracts studied.

**Figure 8:**
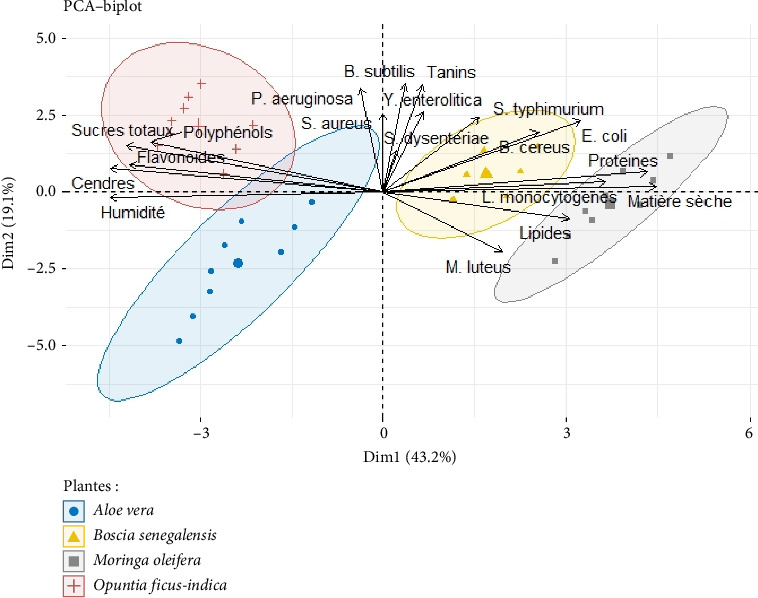
Grouping of inhibition diameters of some bacterial strains according to the phytocompounds of the extracts studied.

PCA of antibacterial activity revealed similarities between the samples analyzed, taking into account the inhibition diameters of the different bacterial strains. PCA (Figure 7) revealed that all the 200 mg/mL extracts had an inhibitory power whose efficacy was almost similar from one extract to another. However, while all 04 extracts showed almost the same degree of efficacy, it was nevertheless observed that the Gram-positive bacteria group was much more sensitive to the action of each extract compared to the Gram-negative bacteria group.  Figure 8 also shows the relationship between bacterial inhibition diameters and the phytocompounds previously evaluated. There is a positive correlation between these phytocompounds and bacterial growth inhibition after application of *M. oleifera*, *B. senegalensis*, *A. vera*, or *O. ficus-indica* extracts.

### 3.2. Discussion

Many studies have indicated that the coagulation or flocculation capacity of plant extracts is strongly linked to the presence of functional groups such as polyphenols, polysaccharides, proteins, and tannins [17]. Thus, the identification and quantification of phytocompounds in the extracts studied showed very interesting results in terms of their biochemical composition and antibacterial properties. *M. oleifera* and *B. senegalensis* seeds showed low water content compared with *A. vera* leaves and *O. ficus-indica* cladodes. The high water content of *A. vera* leaves and *O. ficus-indica* cladodes is partly explained by the water storage role played by these plants [18, 19]. Indeed, these two plants have the capacity to retain large quantities of water, which can fluctuate between 80% and 95% of their total mass. This capability makes it a promising source of natural hydrocolloids, offering significant thickening and gelling effects for applications in the chemical and cosmetics industries [20]. Ash content was also higher in *A. vera* leaves and *O. ficus-indica* cladodes than in *M. oleifera* and *B. senegalensis* seeds. This variation is attributable to the metabolism of each plant, the nature of the soil and the climate, as well as to genetic factors [21]. This study also enabled us to assess the content of secondary metabolites, confirming that *M. oleifera* seeds, *B. senegalensis* seeds, *A. vera* leaves, and *O. ficus-indica* cladodes contain significant quantities of polyphenols, flavonoids, and tannins. However, an objective comparison of the results of this study with those of the literature is difficult due to the influence of various factors specific to each plant, such as genetic factors, degree of ripening, climatic and environmental conditions, extraction, and dosage methods [22, 23]. Some authors [21, 24] support this approach, asserting that the accumulation of secondary metabolites in plants depends on biotic and abiotic factors and is reflected in the fluctuation of their content from one plant to another. However, the results of this study fall within the range of values reported by various authors: [25] for *M. oleifera* seeds, [26] for *O. ficus-indica* cladodes, [27] for *A. vera* leaves, and [28] for *B. senegalensis* seeds.

Moreover, the importance of secondary metabolites in water treatment through coagulation and flocculation using plant extracts has been highlighted by several authors who demonstrated the role of polyphenols and flavonoids in the purification of unsafe water [29, 30].

Tannins have also been shown to have very interesting capacities in terms of abating water turbidity during clarification [17]. According to some studies, the chemical structure of tannins interacts with the suspended particles present in raw water [31]. The hydroxyl groups (OH) of the phenol groups present in the tannin structure give it an anionic character, thereby helping to improve the sanitary quality of water after coagulation/flocculation treatment [32].

The analytical results of this study also enabled the identification and quantification of primary metabolites such as total sugars, proteins, and lipids, which would also be involved in coagulation/flocculation during the application of bioextracts in water treatment. This study reported total sugar contents ranging from 6.2 to 35.52 ± 1.63 g/100 g DM, with the highest levels recorded for *O. ficus-indica* cladodes and *A. vera* leaves. This can be explained by the fact that these 02 plants are mucilaginous species, mucilages being complex polymers of a carbohydrate nature with a strong branched structure [33]. According to some studies, the mucilages of *A. vera* and *O. ficus-indica* contain significant amounts of sugars in polymeric forms, such as cellulose and starch, or in monomeric forms [34]. The mucilage of *O. ficus-indica* has been reported to include D-glucose, D-galactose, arabinose, D-xylose, and L-rhamnose [35]. Additionally, these sugars have been shown to play a role in the removal of turbidity from raw water during coagulation and flocculation processes [36, 37].

As for protein content, the present study reported values ranging from 0.85 to 34.5 ± 1.53 g/100 g, the highest of which was recorded for *M. oleifera* seeds. In fact, several authors have reported that *M. oleifera* seeds are an important source of protein whose capacities could be put to good use in raw water treatment [38, 39]. Studies have shown that M. oleifera seeds contain a basic polypeptide composed of active cationic polyelectrolytes. These polyelectrolytes are capable of neutralizing colloids in unsanitary water, as most colloids are negatively charged [40]. This polypeptide is soluble in polar solvents such as water, ethanol, and methanol. [41].

As for fat content, our results showed that only *M. oleifera* seeds represent a significant source of fat (21.4 ± 0.85 g/100 g MS). According to some studies, similar values were reported [42–44].

A study of the antibacterial activity of *M. oleifera*, *B. senegalensis*, *O. ficus-indica*, and *A. vera* extracts showed good potential for inhibiting Gram-positive and Gram-negative bacterial strains. At 100 mg/mL, the majority of inhibition diameters ranged from 8.00 ± 1.73 to 9.67 ± 0.58 mm. This indicates that at this concentration, each of the four extracts has a low antibacterial activity, given that the diameter of the disks was 6 mm. As a result, by increasing the concentration of the extracts to 200 mg/mL, the inhibition diameters varied between 12.00 ± 1.00 and 16.33 ± 1.53 mm. Antibacterial activity was found to be more pronounced against Gram-positive bacteria than Gram-negative bacteria. These results are similar to those obtained in other studies [45, 46], which also report that plant extracts have a better potential against Gram-positive bacteria than Gram-negative bacteria. In fact, Gram-positive and Gram-negative bacteria differ in structure. The outer membrane that surrounds the cell wall is found only in Gram-negative bacteria, and would be highly impermeable to the passage of substances inside the bacteria, unlike Gram-positive bacteria [47].

It should also be noted that most of the strains tested are food poisoning bacteria, which is of vital importance in the food industry. Furthermore, the antimicrobial capacity of plants is thought to be due to the presence of secondary metabolites [48, 49]. Some authors [15, 50] report that phenolic compounds have high antibacterial power and that the antimicrobial potential of plants is due to the various secondary metabolites, namely phenols, flavones, flavanols, alkaloids, and many others.

## 4. Conclusion

The study of the biochemical composition and antimicrobial activity of *M. oleifera* seeds, *B. senegalensis* seeds, *A. vera* leaves, and *O. ficus-indica* cladodes has produced promising results. *M. oleifera* and *B. senegalensis* seeds contain a variety of bioactive compounds, including polyphenols, flavonoids, tannins, and proteins, which have demonstrated strong antimicrobial activity against various pathogenic microorganisms. In addition, *A. vera* leaves and *O. ficus-indica* cladodes were identified as mucilaginous plants rich in carbohydrates, possessing properties that would facilitate the removal of particles suspended in contaminated water. Overall, all four extracts showed antimicrobial activity against pathogenic bacteria, underlining their potential as cost-effective and sustainable solutions for providing drinking water in resource-limited settings. However, one of the main limitations of the study is the lack of determination of minimum inhibitory concentration (MIC) and minimum bactericidal concentration (MBC), which are essential to accurately assess their antibacterial potency. Future research should aim to fill this gap, while exploring the stability and scalability of these extracts for practical water treatment applications.

## Figures and Tables

**Table 1:** Sensibility of bacterial strains.

Sensibility	Inhibition zone (mm)
Not-sensitive or resistant	Diameter < 8
Sensitive	Diameter between 9 and 14
Very sensitive	Diameter between 15 and 19
Extremely sensitive	Diameter > 20

**Table 2:** Characterization of biochemical families.

Chemical groups	*Moringa oleifera*	*Boscia senegalensis*	*Aloe vera*	*Opuntia ficus-indica*
Polyphenols	++	+++	++	+++
Flavonoids	+	++	+	++
Tannins	+	+	+	++
Alkaloids	+	+	−	+++
Saponosides	−	+++	++	+
Steroids/terpenes	+	++	+	++

*Note:* (−): absent; (+): weakly present; (++): moderately present; (+++): strongly present.

**Table 3:** Inhibition diameters of extracts studied on Gram-positive bacteria.

Extract	Concentration (mg/mL)	*Bacillus cereus*	*Bacillus subtilis*	*Listeria monocytogenes*	*Micrococcus luteus*	*Staphylococcus aureus*
*M. oleifera*	100	8.50 ± 1.00^bcd^	8.67 ± 0.58^c^	9.00 ± 1.73^c^	8.33 ± 2.08^cd^	8.67 ± 0.58^b^
	200	15.00 ± 2.00^a^	12.33 ± 1.53^ab^	12.00 ± 1.73^a^	13.67 ± 0.58^a^	12.00 ± 1.00^a^

*B. senegalensis*	100	8.00 ± 1.73^d^	9.67 ± 1.53^bc^	9.33 ± 1.15^bc^	ND	ND
	200	14.33 ± 2.08^cd^	13.33 ± 0.58^c^	14.00 ± 1.00^c^	14.33 ± 2.08^d^	13.00 ± 1.00^b^

*A. vera*	100	9.00 ± 2.65^cd^	9.67 ± 1.53^c^	8.33 ± 2.31^c^	9.00 ± 2.00^cd^	ND
	200	15.00 ± 2.65^abc^	14.33 ± 1.15^ab^	16.33 ± 1.53^ab^	16.00 ± 2.00^ab^	14.67 ± 1.53^a^

*O. ficus-indica*	100	8.67 ± 1.15^cd^	8.50 ± 2.65c	8.67 ± 1.15^c^	8.50 ± 2.65^bcd^	9.67 ± 1.53^b^
	200	15.33 ± 2.08^ab^	16.00 ± 2.00^a^	13.67 ± 2.31^ab^	13.00 ± 3.46^abc^	13.67 ± 2.89^a^

*Note:* In each column, values sharing the same letter are not significantly different according to the Tukey's HSD test at the 5% level.

**Table 4:** Inhibition diameters of extracts studied and gentamicin on Gram-negative bacteria.

Extract	Concentration (mg/mL)	*Escherichia coli*	*Pseudomonas aeruginosa*	*Salmonella typhimurium*	*Shigella dysenteriae*	*Yersinia enterocolitica*
*M. oleifera*	100	8.67 ± 1.53^b^	8.00 ± 2.08^b^	8.33 ± 0.58^c^	8.67 ± 0.58^c^	8.67 ± 1.53^ab^
	200	14.67 ± 2.08^a^	14.33 ± 2.52^a^	13.33 ± 1.53^a^	14.00 ± 2.08^a^	13.67 ± 3.79^a^

*B. senegalensis*	100	8.33 ± 1.53^b^	8.00 ± 1.00^b^	7.33 ± 1.73^bc^	8.00 ± 1.00^bc^	8.33 ± 0.58^ab^
	200	13.67 ± 0.58^a^	12.67 ± 0.58^a^	11.67 ± 1.00^a^	12.33 ± 2.08^a^	14.33 ± 0.58^a^

*A. vera*	100	9.67 ± 1.15^b^	9.00 ± 1.00^b^	8.33 ± 1.15^c^	9.33 ± 0.58^c^	ND
	200	14.33 ± 1.53^a^	14.00 ± 1.00^ab^	14.33 ± 1.53^ab^	12.33 ± 1.53^ab^	13.67 ± 2.52^a^

*O. ficus-indica*	100	7.67 ± 2.00^b^	8.67 ± 2.08^b^	8.33 ± 2.31^c^	8,33 ± 1.53^c^	7.67 ± 0,58^ab^
	200	13.67 ± 0.58^a^	14.33 ± 0.58^a^	14.00 ± 1.00^a^	14.33 ± 1.53^a^	13.33 ± 2.52^a^

Gentamicin	200	28.33 ± 1.52	31.33 ± 1.15	29.12 ± 1.02	41,33 ± 1.52	35.67 ± 1.15

*Note:* In each column, values sharing the same letter are not significantly different according to the Tukey's HSD test at the 5% level.

## Data Availability

The data used in this research have not been previously published or made publicly available in any form. The datasets used and/or analyzed in this study are available on request from the corresponding author.
